# Parental involvement and student creativity: a three-level meta-analysis

**DOI:** 10.3389/fpsyg.2024.1407279

**Published:** 2024-09-11

**Authors:** Huiyong Fan, Yuxiang Feng, Yichi Zhang

**Affiliations:** College of Educational Science, Bohai University, Jinzhou, China

**Keywords:** parental involvement, creativity, students, meta-analysis, three-level modeling

## Abstract

**Introduction:**

The Ecological Systems Model of Creativity Development (ESMCD) proposes that parental involvement positively impacts student creativity. However, prior empirical studies present mixed results, including positive, negative, and no correlations between these variables.

**Methods:**

To synthesize these inconsistent primary studies, the current study conducted a systematic meta-analysis synthesizing 30 primary studies involving 37 independent samples with 70 effect sizes and a total *N* = 20,906 participants.

**Results:**

The results demonstrated: (1) an overall significant small, positive correlation (*r* = 0.101) between parental involvement and student creativity; (2) significant small, positive correlations between specific involvement types (autonomy support *r* = 0.144; behavioral control *r* = 0.133; content support *r* = 0.131) and creativity, alongside a significant small, negative correlation between psychological control and creativity (*r* = −0.117); (3) no statistically significant moderating effects of student grade level, parental gender, region, or publication type.

**Discussion:**

This systematic meta-analytic review consolidates empirical evidence indicating that parental involvement positively predicts students’ creativity, while highlighting the detrimental impact of psychological control on creative outcomes. Further research elucidating the mechanisms underlying these relations is critical for informing parenting approaches and education policies seeking to foster creativity development among students.

## 1 Introduction

Creativity, defined as the capacity to generate products that are novel, socially valuable, and appropriate (Zhang and Sternberg, 2001; Runco and Jaeger, 2012; Sternberg et al., 2024), constitutes a pivotal engine of societal advancement.

Parental involvement is defined as the active participation of parents in all aspects of their children’s social, emotional, and academic development (Castro et al., 2015), which has a multifaceted and multidimensional inner structure (Epstein, 1990, 1995; Jeynes, 2007; Pomerantz et al., 2007).

Studies probing the link between parental involvement and learning outcomes can be traced back to 50 years ago (e.g., Coleman, 1966, 1968; Epstein, 1990, 1995; Fiskerstrand, 2022; Kim, 2022). However, the existent empirical findings leave some issues still unsolved. Firstly, the results about the relationship between parental involvement and creativity were inconsistent which could be divided into three categories, some suggested a positive correlation, others reported a negative correlation, and still others suggested no correlation. Secondly, some empirical findings were inconsistent with the prediction deduced from existent theory. The Ecological Systems Model of Creativity Development (ESMCD) argued that parental involvement was conducive to fostering creativity. The negative, or insignificant correlations were not consistent with the theoretical prediction. ESMCD postulated that parents could provide guidance when children were in the process of problem-solving, parents could also provide encourage independence and autonomy, supporting their children’s decision-making with the parent-child relationships which played a key role in promoting creative development (Yeh, 2004).

The present paper provides a synthesis about the existent inconsistent results using a three level meta analysis, clarify the link between empirical evidence and theoretical research, and identify some issues need to be studied in the future.

### 1.1 The importance of creativity

The cultivation of creativity among students holds profound significance for both individual development and national prosperity (Pang and Plucker, 2012).

First, creativity is vital to individual development. The OECD (2020) conducted a study to identify the key factors for personal success in the 21st century, and found that creativity is a key factor (Collard and Looney, 2014). A meta-analysis showed that creativity is associated with individual success outcome indicators such as problem solving procedures, prestige of honors or academic awards, working environments, class climate, openness to new experiences, affective sensibility and leadership (Ma, 2009).

Second, creativity is closely related to national prosperity. Creativity is the source of innovation which is a driving force of economic and social progress. As for delivering sustainable economic growth and social development, creativity have a greater effect than the traditional inputs such as labour or capital (Bobirca and Draghici, 2011). Consequently, the ability of any state to attract, retain and develop creative human capital and to exploit creative capabilities tends to become, to a significant extent, the key to global competitiveness.

### 1.2 Research on parental involvement

#### 1.2.1 The construct of parental involvement

There are two categories defining parental involvement: the static structure view and the dynamic process view.

The static structure view suggested that parental involvement was divided into several specific types. Epstein (1995) presented there are six types of parental involvement including parenting, communication, volunteering, learning at home, decision making, and collaborating with the community.

Similarly, Xu et al. (2024) reviewed about 30 primary studies, and found that parental involvement in the homework field can be divided into five types, including autonomy support, content support, parental control, frequency, and mixed. Autonomy support mean parents attend to students’ ideas and support their homework initiatives. Content support mean parents provide direct support on the content of assignments. Parental control mean when parents function to monitor, control, and interfere with homework assignments. Parental involvement was coded as “frequency” when it focused on the frequency or amount of its involvement. A study was coded as “mixed” when it included more than one dimension of involvement.

The dynamic process view holds that parental involvement as a whole is a dynamic process (Fiskerstrand, 2022). Fiskerstrand (2022) classified parental involvement into 12 categories, including competence, belief, motivation, emotion, presence, framing, parenting, activity, talking, helping, choosing, and outing. From the perspective of being, doing, and thinking, these 12 categories can be grouped into four classes. They are the parental thinking (including competence, belief, motivation, emotion), the parental being (including presence, framing, parenting), and the parental doing (including activity, talking, helping, choosing, outing) (Fiskerstrand, 2022).

Considering the primary studies included, the present project adopted the Xu et al. (2024) framework to classify parental involvement. Then the parental involvement were divided into four subtypes: autonomy support (AS), parental control (PC), behavior control (BC) and content support (CS).

#### 1.2.2 Relevant studies on the outcomes of parental involvement

Previous studies mainly focused on parental involvement and academic achievement, schooling and children’s adjustment (including truancy control, STEM learning, reading, computational thinking), student engagement (including home-based digital learning), and resilience.

Some studies have explored the relationship between parental involvement and academic achievement, and found that parental involvement can promote academic achievement. Wilder (2014) synthesized the results of nine meta-analyses and the results indicated that the relationship between parental involvement and academic achievement was positive, regardless of a definition of parental involvement or measure of achievement. Similarly, Kim (2022) has conducted a second-order meta-analysis of parental involvement and achievement research over the past 50 years (including 1,177 primary studies in 23 meta-analyses), the results show that there is a positive association between parental involvement and achievement. For the impact of specific types of parental involvement, Kim (2022) and Wilder (2014) had consistent results, pointing to the strongest effect for parent expectations and aspirations and mixed results for homework help. The meta-analysis results of Jiang et al. (2023) shows an overall positive link between supportive parent homework involvement and students’ mathematics achievement and a negative link between intrusive parent homework involvement and students’ mathematics achievement. Xu et al. (2024) used three-level meta-analysis to explore the relationship between parental homework involvement and students’ achievement. The results revealed an overall weak negative relationship between parental homework involvement and students’ achievement. Within specific categories of parental involvement, students’ achievement was positively related to autonomy support, but largely unrelated to content support, parental control, frequency, and mixed.

Some studies have studied the relationship between parental involvement and schooling and children’s adjustment (including truancy control, STEM learning, reading, computational thinking) and revealed positive association between parents’ involvement in children’s schooling and children’s adjustment. The meta-analysis by Barger et al. (2019) revealed small positive associations (*r* = 0.13 to 0.23) between parents’ naturally-occurring involvement in children’s schooling and children’s academic adjustment (i.e., achievement, engagement, and motivation) that were maintained over time. Parents’ involvement was also positively related to children’s social (*r* = 0.12) and emotional adjustment (*r* = 0.17) and negatively related to their delinquency (*r* = −0.15), concurrently. Kanungo et al. (2024) conducted a scoping review analysis (including 17 articles) of the forms of parental involvement for truancy control, the findings indicated that volunteering and communication are adequate indicators of truancy control and both indicators can significantly impact truancy prevention programmes. The review of Thomas et al. (2020) about parent involvement and its influence on children’s STEM learning found that parent involvement positively affects children’s quantitative skills and problem-solving skills in the terms of the specific skills in STEM content areas.

In addition, some studies have found that parental involvement also promotes students’ reading (Çaliskan and Ulas, 2022) and computational thinking (Cai and Wong, 2023). Çaliskan and Ulas (2022) employed a pretest-posttest quasi-experimental design with a paired control group, data were collected from a total of 100 fourth graders studying in two different primary schools. The findings showed that there were significant differences between the experimental group and the control group, meaning that the parent-involved reading activities developed by the researchers had a positive effect on the students’ reading comprehension, reading motivation, and attitudes towards reading. Cai and Wong (2023) conducted a systematic review about parental involvement in computational thinking education found that three ways in which parents were involved (including affective, behavioral, and cognitive participation) all play an active role in students’ computational thinking education.

Some studies have tapped the relationship between parental involvement and student engagement and revealed there was a positive correlation between parental involvement and student engagement (home-based digital learning belongs to its subcategory). Erol and Turhan (2018) investigated 1,488 students and used the Parental Involvement Scale and Engagement to School Scale to measure parental involvement and student engagement. The results showed a significant positive correlation between parental involvement and student engagement. Increasing and encouraging parental participation in the educational process can enhance students’ engagement with the school. A review of Yang et al. (2023) revealed that parental involvement was the most crucial aspects of social support for students’ school engagement. The results also found that student engagement was reflected via ABC dimensions (i.e., affective, behavioural, and cognitive). Qualter (2024) conducted a narrative literature review about shaping the role of parental involvement in home-based digital learning found that parental involvement plays the most central role in home-based digital learning. The enhancement of parents’ self-efficacy and motivation in home-based digital learning is conducive to the development and education of children’s digital technologies.

Some studies have discussed the relationship between parental involvement and resilience and revealed parental involvement has a positive impact on student engagement. Kovács et al. (2022) conducted a systematic literature review about resilience of parental involvement found that whether the goal is to build upon resilience as a personality trait or target its development as a consequence, strong collaboration between the parents, teachers and professionals concerned in the process can significantly contribute to the child’s psychological, emotional and academic development. Olaseni (2020) conducted an empirical study with a sample of 347 adolescents in Nigeria and found that parental involvement significantly predicted academic resilience in such a way that high parental involvement is linked with high academic resilience. Cui et al. (2024) studied 105,641 Chinese students using latent profile analyses (LPAs) and multivariate analysis of covariance (MANCOVA) and found that parents’ more frequent involvement in students’ everyday lives, coupled with less frequent involvement in their study matters, may effectively foster academic success and enhance the development of resilient traits.

### 1.3 The relationship between parental involvement and creativity

Three perspectives have been proposed by previous empirical research on the relationship between parental involvement and student creativity: the facilitating view, the hindering view, and the non-correlation view.

The facilitating view proposes parental involvement promotes creative development, reflected by significant positive correlations. Niu (2007) conducted a study on Chinese high school students, and found autonomy-supportive parenting positively predicted creativity. Likewise, Pugsley and Acar (2020) surveyed 1,324 parents, in which the assessment of children’s creativity was reported by parents, identified creative home environments and values were associated with higher children’s creativity.

Conversely, the hindering view argues parental involvement impedes creativity, with significant negative correlations. Jankowska and Karwowski (2019) surveyed 75 elementary school students and found a negative correlation between parental performance-orientation and creative thinking in elementary students. Similarly, Krumm et al. (2013) found disciplinary and overinvolved parenting related to lower creativity.

Finally, the non-correlation view shows no direct link between parental involvement and student creativity. Oh et al. (2014) surveyed 137 primary school students and found maternal involvement unrelated to primary students’ creativity score based on the science creativity test (SCT) scale. Likewise, Guo et al. (2021) surveyed 559 students and evidenced personality traits (openness and darkness) fully mediated the relationship between parental involvement and creativity, and the correlation between parental involvement and creativity was close to zero.

### 1.4 The research questions and hypotheses of the present study

Despite emerging attention to the parental involvement-creativity link since the 1990s, empirical findings and theoretical predictions remain inconsistent. To integrate these contradictory results, the present study employs a three-level meta-analytic model (Assink and Wibbelink, 2016; Cheung, 2014) synthesizing the literature. The research objective is to examine the association between parental involvement and student creativity alongside moderating factors impacting this association. Specifically, this meta-analysis addresses the two-fold research questions coming as follows:

(1) What is the relationship between parental involvement and student creativity (positive, negative or no correlations)?
(2) Do the following variables moderate this relationship: involvement type (autonomy support, psychological control, behavioral control, content support), grade level, parental gender, region, and publication status?

#### 1.4.1 The relationship between parental involvement and creative relationships

Previous findings have yielded three perspectives: the facilitating view, hindering view, and non-correlation view. Bronfenbrenner’s early theory (Bronfenbrenner, 1977; Bronfenbrenner and Morris, 2007; Woolfolk, 2013) broadly proposed environmental influences on development without specifying directionality. Yeh (1999, 2004) delineated four positive parental involvement types promoting creativity through “appropriate family climate,” “creative activity experience,” “creative skill guidance” and “appropriate parenting styles.” However, additional involvement styles exist, including psychological control and detachment. When examined holistically using omnibus measurements, the cumulative role of parental involvement should demonstrate a blended influence across facilitating, hindering and null effects. Therefore, we propose the following opening hypothesis:

*Hypothesis 1*. Parental involvement and creativity will demonstrate a significant but small correlation of less than 0.200.

#### 1.4.2 Potential moderators

The relationship between parental involvement and student creativity in the following studies may have been moderated by several variables: parental involvement type, grade, parental gender, region, and publication type.

#### 1.4.3 Parental involvement type

Parental involvement can be described using different models. From the measure perspective, concerning, parental involvement can be divided into four subtypes: autonomy support (AS), parental control (PC), behavior control (BC) and content support (CS) (Xu et al., 2024).

AS refers to respecting children’s perspectives, encouraging independent problem-solving, providing choices (Grolnick and Ryan, 1989; Ryan et al., 2006). Tang et al. (2022) surveyed on 5,523 students and evidenced AS positively predicted elementary/middle schoolers’ creativity (*r* = 0.100). Chen et al. (2021) investigated a sample of 258 7th graders and revealed a significant correlation between parental autonomy support and creative thinking (*r* = 0.200).

Alternatively, PC entails coercive, psychologically intrusive tactics inducing compliance via love withdrawal or guilt, it often divided into psychological control (PyC) and behavioral control (BC). PyC encompasses parental intrusion into the child’s inner world, aiming to align the child’s thoughts, behaviors, and feelings with parental demands through strategies such as withdrawal of love and guilt induction, whereas BC regulates behavior through monitoring and discipline to social norms (Barber, 1996; Barber and Harmon, 2002; Silk et al., 2003). Chen et al. (2021) found that BC dimensions positively (*r* = 0.270, 0.320 and 0.200), and PC dimensions negatively (*r* = −0.080, −0.070 and −0.060) correlated with the three dimension of creative thinking (fluency, flexibility, and originality). Ren et al. (2017) found that BC dimensions positively (*r* = 0.110, 0.110 and 0.100), and PC dimensions negatively (*r* = −0.100, −0.100 and −0.040) correlated with the three dimension of creative thinking.

Finally, CS represents direct academic involvement like guidance and home-school communication (Cho and Lin, 2011; Oh et al., 2014; Zheng et al., 2020) linked CS to heightened creativity (*r* = 0.290) on the creative problem solving in math/science scale among 733 elementary/middle schoolers (Cho and Lin, 2011).

These differential effects align with self-determination theory positing autonomy, relatedness, and competence as undergirding motivation and creativity (Ryan and Deci, 2019, 2020). In contrast, parental psychological control, such as “love recycling” and “guilt triggering,” thwarts intrinsic motivation, narrowing exploratory behavior and hampering creativity. Accordingly, we propose the Hypothesis 2.

*Hypothesis 2*. The moderating effect of parental involvement subtype will be significant.

#### 1.4.4 Grade

The parental involvement-creativity link may vary by children’s grade level. As grade level increases, opportunities for involvement in creative development may diminish for several reasons: increasing academic difficulty and specialization exceeding parental expertise, long times of school life and study limiting parent-child engagement, and children’s advancing cognitive skills (Said-Metwaly et al., 2021).

Empirically, moderating effects of grade have been demonstrated. Cho and Lin (2011) surveyed 733 elementary and middle school pupils and found positive family processes more strongly correlated with creative problem-solving in elementary than middle or high schoolers. Liang et al. (2021) also showed grade level differences in the involvement-creativity relationship for both mini-c and little-c creativity among 526 students. Collectively, these results indicate parental involvement exerts greater impact at elementary relative to secondary levels as academic contexts shift. Therefore, we propose Hypothesis 3.

*Hypothesis 3*. The moderating effect of grade level on the parental involvement-creativity relationship will be significant.

#### 1.4.5 Parental gender

Theoretically, both paternal and maternal involvement could encompass autonomy support, psychological control, behavior control and content support. However, differential links with creativity have been proposed. For instance, among 65 5th graders, Wallinga and Crase (1979) evidenced significant positive father-creativity but not mother-creativity correlations. Yet with 550 Chinese high schoolers, Liu et al. (2013) found maternal involvement more strongly favored creative thinking development. Due to the inconsistency among various research, we propose Hypothesis 4:

*Hypothesis 4*. The moderating effect of parental gender on the parental involvement-creativity relationship will not reach statistical significance.

#### 1.4.6 Region

The influence of region on the interplay between parental involvement and student creativity warrants examination. Regions are often distinguished as Eastern (e.g., Chinese, Korean, and Japan), associated with psychological control and content support, and Western (e.g., European countries such as England and France and their former colonies) emphasizing autonomy support and behavioral monitoring (Niu and Sternberg, 2003; Ofole and Ezeokoli, 2014; Oh et al., 2014; Man et al., 2015; Fanchini et al., 2018; Zhang, 2023).

Eastern culture may have a dampening influence on creativity. Niu and Sternberg (2001), through the experimental study of students from two different cultures in China and the United States, showed that the artistic creativity of Chinese students was more likely to be reduced as a function of restrictive task constraints or of the absence of explicit instructions to be creative.

Western culture may have a stimulating influence on creativity. Niu and Sternberg (2001) showed that an independent self-oriented culture (western culture) is more encouraging of the development of artistic creativity than is an interdependent self-oriented culture (eastern culture). Shao et al. (2019) found that individuals from different cultures, particularly those from individualist and collectivist cultures, show differences in preferred creative processes and creative processing modes. To be specific, usefulness seems more important than novelty in the East, whereas novelty seems equally important as usefulness, if not more so, in the West when they are engaged in creative endeavors.

These varying socialization patterns cultivate differing levels of creativity across cultures (Niu and Sternberg, 2001). Overarching cultural orientation effects also emerge empirically. For example, in a cross-cultural study across eight countries, Zhang et al. (2021) found the involvement-creativity link varied significantly, with nonsignificant correlations in most Eastern countries (China, Kosovo, Russia, Saudi Arabia) in contrast to a significant negative correlation in the Western country of Chile. Therefore, we hypothesize:

*Hypothesis 5*. The moderating effect of region on the parental involvement-creativity relationship will be significant.

#### 1.4.7 Publication type

According to previous research (Card, 2012), publication type may also be a potential moderating variable. During journal review processes, core publications preferentially select significant findings over nonsignificant results published in peripheral outlets like dissertations or conference papers (Borenstein et al., 2009; Card, 2012). Our literature review observed such patterns; for example, Yin (2019) found a nonsignificant parental involvement-creativity association in a master thesis. By contrast, among studies published in core educational psychology journals, like Man et al. (2015) in a journal found that parental behavioral control and psychological control and social creativity were significantly correlated with correlation coefficients of 0.090 and −0.210. This expected effect of publication bias leads to our final hypothesis:

*Hypothesis 6*. The moderating influence of publication type on the parental involvement-creativity relationship will be statistically significant.

**Figure 1:**
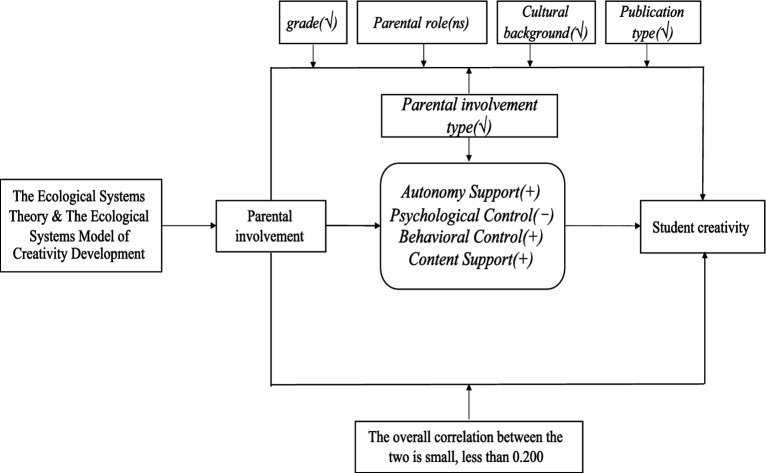
Research hypothesis of the present study. “+” indicates a positive effect, “−” indicates a negative effect, “√” indicates a significant moderating effect, “ns” indicates a non-significant moderating effect.

All research hypotheses are organized as a diagram as shown in Figure 1.

## 2 Methods

### 2.1 Literature search

#### 2.1.1 Scope and modalities of the search

**Figure 2:**
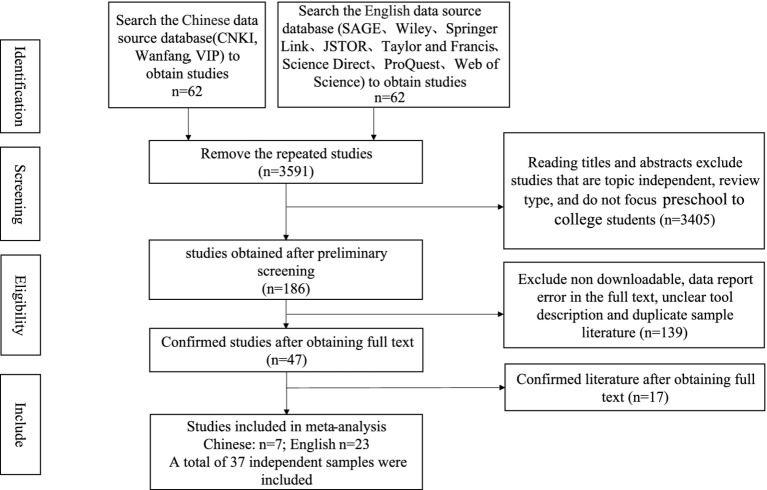
The search process and search results of the present study.

A systematic search was conducted across major Chinese (Chinese National Knowledge Infrastructure (CNKI), Wanfang Data Knowledge Service Platform, VIP Chinese Journal Service Platform) and English (SAGE, Wiley, Springer, Taylor and Francis, ScienceDirect, ProQuest, JSTOR, Web of Science) databases combining parental related terms (“parental,” “paternal,” “maternal”) with involvement terms (“involvement,” “assistance”) and creativity terms (“creativity,” “creative thinking”) in title, abstract and keyword fields. To minimize publication bias, additional searches utilized public search engines and reference lists, including relevant reviews (e.g., van der Zanden et al., 2020). The final search date was November 6, 2023 resulting in an initial pool of 3,777 records. Figure 2 diagrams the systematic search and selection process.

#### 2.1.2 Inclusion and exclusion criteria

Studies that met the following criteria were included in the meta-analysis: (1) reported correlation coefficients between the total score or dimensions of parental involvement and student creativity and its sub-dimensions, or other statistics that could be transformed into correlation coefficients, such as *F*-values or *beta* values. Multiple regressions were excluded due to difficulty in recovery. (2) The study population consisted of students from kindergarten through undergraduate level. Primary means K-6, middle school and high school mean 7–12. (3) Sample sizes for independent samples are reported. (4) Measurement instruments for and parental involvement and creativity were reported.

Studies that met the following criteria were excluded in the meta-analysis:

(1) Creative self-efficacy and creativity are not the same concept, and related literature is not included in this meta-analysis.
(2) Since parenting style and parental involvement are not the same concept, related literature is also excluded.

#### 2.1.3 Search results

More than 40 studies were initially retrieved, and a total of 30 studies met the above criteria (see Table 1), containing 37 independent samples, with a total participation of 20,906, including 7 Chinese-language studies, 23 English-language studies, 24 journal articles, and 6 dissertations. The publication years span from 1991 to 2022.

**Table 1:** Basic information of the primary studies included in this meta-analysis.

Author, year^a^	Effect size number	Sample size	Parental involvement type^b^	Female ratio	Grade	Country	Measurement tools of creativity^d^	Parental role	Publication type^e^	Quality assessment
Chagas, 2009	1	28	CS	0.5	Primary and middle	Portugal	TCT-DP	Parents	J	15
Chen, 2021	3	258	AS/PyC/BC	0.48	Middle	China	rCAB	Parents	J	18
Cho and Lin, 2011	1	733	CS	NA^c^	Primary and middle	Korea	CPSMS	Parents	J	20
Cho and Lin, 2011	1	236	CS	NA	Primary	Korea	CPSMS	Parents	J	20
Cho and Lin, 2011	1	328	CS	NA	Middle	Korea	CPSMS	Parents	J	20
Cho and Lin, 2011	1	169	CS	NA	Middle	Korea	CPSMS	Parents	J	20
Cho and Lin, 2011	1	24	CS	NA	Primary	Korea	CPSMS	Parents	J	20
Cho and Lin, 2011	1	22	CS	NA	Middle	Korea	CPSMS	Parents	J	20
Diarra, 2017	2	262	AS/BC	0.62	Primary	Mali	SCMQ	Parents	J	13
Fanchini, 2018	1	855	CS	0.48	Primary	France	Self-report	Parents	J	15
Gralewski, 2020	2	552	AS/PyC	0.59	Middle	Poland	TCT-DP	Mother	J	17
Guo, 2021	2	559	AS/BC	0.68	College	China	AUT, ICAA	Parents	J	16
Jankowska, 2019	2	75	CS	0.55	Primary	Poland	TCT-DP	Parents	J	17
Kim, 2020	2	333	CS	0.51	Primary and middle	Korea	TTCT	Parents	J	15
Kong, 2019	1	417	AS	0.51	Preschool	China	CPTC	Parents	D	20
Krumm, 2015	1	359	CS	0.54	Primary and middle	Argentina	TTCT	Parents	J	14
Li, 2021	2	776	AS/PyC	0.49	Middle	China	MDCBQ	Parents	D	20
Liang et al., 2021	3	80	AS/PyC/CS	0.48	Middle	China	TTCT, CAT, CDQ-R	Parents	J	15
Liang et al., 2021	5	526	AS/PyC/CS	NA	Primary and middle	China	Absi, CDQ—R, CSE	Father/mother/parents	J	17
Liu, 2013	4	550	AS/CS	0.55	Middle	China	TTCT	Father/mother	J	16
Man, 2015	2	540	PyC/BC	0.5	Primary	China	SCT^1^	Father	J	14
Michel, 1991	1	30	CS	0.5	Primary	Britain	TTCT	Mother	J	14
Ofole, 2014	1	677	CS	0.42	Middle	Nigeria	SPB	Parents	J	17
Oh et al., 2014	2	39	CS	0.33	Primary	Korea	SCT^2^	Father/mother	J	15
Oh et al., 2014	2	98	CS	0.56	Primary	Korea	SCT^2^	Father/mother	J	15
Pérez-fuentes,2019	4	742	AS/PyC/BC/CS	0.53	Primary and middle	Spain	CBQD	Parents	J	15
Pugsley,2020	3	962	AS/CS	NA	Na	36 countries^f^	TICC	Parents	J	16
Ren, 2017	2	503	PyC/BC	0.49	Middle	China	rCAB	Parents	J	14
Szechter, 2004	1	15	CS	0.67	Preschool	NA	SGM	Parents	J	15
Tang, 2022	1	5,523	AS	0.48	Primary and middle	China	CIB	Parents	J	18
Wang, 2011	4	651	AS/CS	0.55	Middle	China	TTCT	Father/mother	D	20
Wang, 2018	2	800	CS	0.51	Middle	China	rCAB	Father/mother	D	19
Wang, 2019	2	82	AS/CS	0.61	Preschool	China	TCAM	Mother	J	15
Wang, 2023	2	680	AS/PyC	0.44	Middle	China	TTCT-Verbal	Parents	J	17
Yin, 2019	2	1,440	CS	0.49	Primary and middle	China	rCAB	Father/mother	D	18
Zhang, 2022	1	584	AS	0.72	College	China	BICB	Parents	J	18
Zhao, 2012	1	398	AS	0.45	Primary	China	WCS	Parents	D	17

a To reduce space, most list only the first author; u1–u6 indicates a study with multiple independent samples.

b AS, parental autonomy support; PyC, parental psychological control; BC, parental behavioral control; CS, content support.

c NA indicates that the paper does not provide the appropriate information.

d TCT-DP, Test for Creative Thinking-Drawing Production; rCAB, Runco creativity assessment battery; CPSMS, Creative Problem Solving in Mathematics and Science Scale; SCMQ, Social Creativity Measure Questionnaire; AUT, Alternative Uses Test; ICAA, Inventory of Creative Activities and Achievements; TTCT, Torrance Tests of Creative Thinking; CPTC, Teacher evaluation questionnaire for creative personality tendency of children; MDCBQ, Middle school students daily creative behavior questionnaire; CAT, Consensus Assessment Technique; CDQ-R, Creativity Domain Questionnaire; Absi, Aurora battery of successful intelligence; CSE, Creative self-efficacy; SCT^1^, Social creativity test; SPB, Success Potential Battery; SCT^2^, Scientific Creativity Test; CBQD, Creative Behavior Questionnaire: Digital; SGM, Spatial-Graphic Measures; CIB, Runco Ideational Behavior Scale; TCAM, Thinking creatively in action and movement; BICB, Biographical Inventory of Creative Behaviors; WCS, Williams Creativity Scale.

e J, Journal; D, Dissertation.

f Including US, Australia, Canada, UK, New Zealand and other countries.

#### 2.1.4 Coding of original study characteristics

Each study was coded according to the characteristics (see Table 1). It is important to note that effect sizes were calculated in terms of independent samples and were coded separately if more than one independent sample was reported in a single study at the same time.

### 2.2 Extraction of potential moderator variables

The basic characteristics of potential moderating variables extracted from primary studies is listed in Table 2.

**Table 2:** Basic statistics of potential moderators included in the current study.

Moderator variable	Category	Independent sample		Effect size	
		*K*1	%	*K*2	%
Parental involvement Type	Autonomy support (AS)	17	29.82	21	30.00
	Psychological control (PyC)	9	15.79	9	12.86
	Behavioral control (BC)	6	10.53	6	8.57
	Content Support (CS)	25	43.86	34	48.57
Grade	Preschool	2	5.56	4	6.15
	Primary	10	27.78	15	23.08
	Middle	10	27.78	26	40.00
	Primary and middle	12	33.33	17	26.15
	College	2	5.56	3	4.62
Parental gender	Father involvement	8	17.78	9	12.86
	Mother involvement	10	22.22	14	20.00
	Parental involvement	27	60.00	47	67.14
Region	East	26	74.29	51	77.27
	West	9	25.71	15	22.73
Publication type	Journal	24	80.00	58	82.86
	Dissertation	6	20.00	12	17.14
Literature quality	Medium	27	72.97	55	78.57
	High	10	27.03	15	21.43

*K1, number of independent samples; K2, number of effect sizes.*

### 2.3 Coding process and coding confidence

The primary studies were coded by the first author and subsequently screened by the third author, demonstrating a Kappa coefficient of 0.965, with a high consistency (Card, 2012). Any disagreement in coding were addressed through discussion and subsequently revised.

### 2.4 Statistical calculations

#### 2.4.1 Calculation of effect sizes

The effect size used for this research is the correlation coefficient *r* (zero-order *r*) (Borenstein et al., 2009). In the calculation process, each *r* value was first converted to the corresponding Fisher’s *Z* score with the formula *Z* = 0.5 × In ($\frac{1+r}{1–r}$). The Fisher’s *Z* values were then converted to calculate the zero-order correlation coefficient *r* with *r* = $\frac{e^{2z}+1}{e^{2z}-1}$ (Borenstein et al., 2009). Where the variance of *Z* is *V_Z_* =$\frac{1\;}{n-3}$, *n* denotes the sample size, and the standard error of *Z* is SEz =$\sqrt{V_{Z}}$. Krumm et al. (2015) reported the *F*-value. Fanchini et al. (2018) reported the *β*-value. $r=\sqrt{\frac{F}{F+dfe}}$ (Card, 2012), *r* = *β* (simple linear regression) (Kim, 2011) which was transformed into an *r*-value to be included in the calculation.

#### 2.4.2 Model selection

The current study employed a three-level random effects meta-analytic model (Assink and Wibbelink, 2016; Harrer et al., 2021) that assumes error at the sample (level 1, participations), outcome (level 2, effect sizes), and study levels (level 3). This approach estimates intra-cluster shared effect sizes through a random effects model, while permitting the same cluster share a same effect.

#### 2.4.3 Homogeneity test

In this study, *I*^2^ and *Tau*^2^ were used to represent the between-study variance. It is suggested that 25, 50, and 75% of *I*^2^could be used as thresholds for low, moderate, and high heterogeneity (Borenstein et al., 2009). When a three-level model is used, the *Tau*^2^ value is equal to the sum of two components, *Tau*^2^ (level 2) and *Tau*^2^ (level 3) (Assink and Wibbelink, 2016).

#### 2.4.4 Publication bias

Publication bias refers to the phenomenon that studies with significant results are more likely to be published (Borenstein et al., 2009). Rosenthal’s Fail safe *N* (*Nfs*), Funnel plot, trim and fill method, Egger’s regression are commonly used to detect publication bias (Borenstein et al., 2009).

#### 2.4.5 Assessment of quality

The Basic Quality Assessment of Primary Study, BQAPS (Xu et al., 2024 press) was used as a quality assessment tool to assess the quality of studies included in the meta-analysis. Based on the scoring criteria, score of 0–6 categorized as low-quality, 7–12 categorized as low- medium level, 13–18 were categorized as medium-high level, and 19–24 were categorized as high quality.

#### 2.4.6 Statistical calculation tools

All statistical calculations were conducted in R (R Core Team, 2022). The metafor package was used for the calculation of the three-level model (Viechtbauer, 2010; Harrer et al., 2021). The meta package was used for the creation of the funnel plots (Assink and Wibbelink, 2016; Balduzzi et al., 2019; Harrer et al., 2021).

## 3 Results

### 3.1 Characteristics of the original literature included

A total of 30 primary studies with 37 independent samples were included (see Tables 1, 2) in the analysis. The main study characteristics extracted and their frequency distributions are shown in Table 2.

### 3.2 Homogeneity test

The *Q*-test indicated significant heterogeneity among the effect sizes (*Q* = 639.949, *p* < 0.001). The total *I*^2^ = 90.843%, which is greater than the critical value of 75%, indicates heterogeneity in the preliminary study, as shown in Table 3. Therefore, further analysis of moderating variables is required to find the possible reasons for the differences in the results of the primary study.

**Table 3:** Main effects of the relationship between parental involvement and student creativity.

*K*2	Fisher’s *Z*/*SE*	Zero-order *r* (95% CI)	*t*	*Tau*^2^/percentage in total variance	
				Level 2	Level 3
70	0.101/0.020	0.101 [(0.061; 0.140)]	5.070	0.016/	0.002/
				77.263	13.580

*K2 = number of validity values. Variance in level 2 = within-study variation; Variance in level 3 = between-study variation; SE = standard error.*

### 3.3 Publication bias test

The trim and fill method showed a total effect of *r* = 0.089 [95% CI (0.053; 0.126)] across all primary studies after the addition of the 3-effects data, which did not differ significantly from the overall correlation coefficient of the previous pooled effect size of *r* = 0.101 [95% CI (0.061; 0.140)] before and after the trim and fill.

**Figure 3:**
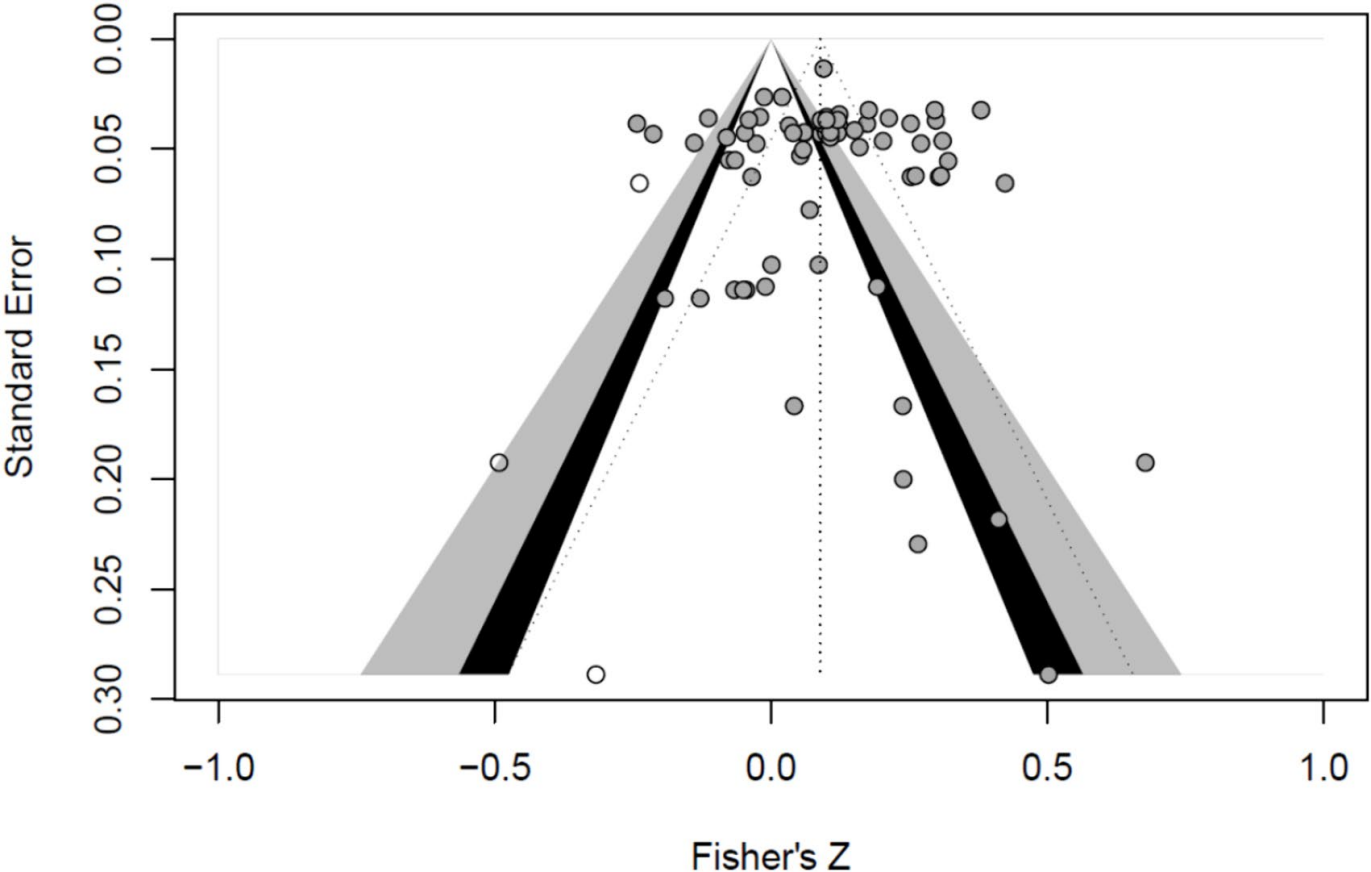
Funnel plot of the distribution of effect values in the present study.

The funnel plot analysis presented that the distribution of effect values is basically symmetric (see Figure 3). The Egger regression with Intercept = 0.085, *SE* = 0.035, *t* = 0.350, *p* = 0.726 indicates that the funnel plot is symmetric. The insecurity coefficient*, Nfs* = 7,386, is greater than 5 *K*1 + 10 = 160. In summary, the effect of publication bias in this paper is negligible.

### 3.4 Moderating effects of literature quality

A meta-regression analysis was conducted with quality score as the independent variable and the effect size value as the dependent variable. The obtained results indicated *F*_(1,68)_ = 1.007; Intercept = −0.067; *SE* = 0.169; *p* = 0.693; *β* = 0.010; *SE* = 0.010; *t* = 1.004; *p* = 0.319. These findings suggest that there is no significant correlation between literature quality scores and effect size values.

### 3.5 Main effects

The correlation between parental involvement and student creativity is *r* = 0.101 [95% CI (0.061; 0.140)], *t* = 5.070, *p* < 0.001 (see Table 3). Following Ellis’s (2010) criteria, the observed relationship between parental involvement and student creativity fell within the category of a low degree of positive correlation.

### 3.6 Moderating effects test

#### 3.6.1 Parental involvement type

The parental involvement type had a significant moderating effect (*F*_(3,66)_ = 17.591, *p* < 0.001) (see Table 4). Autonomy support was positively associated with student creativity with an *r*-value of 0.144 [95% CI (0.092, 0.194)]. The content support group had an *r*-value of 0.131 [95% CI (0.084, 0.179)]. The *r*-value for the psychological control group was −0.117 [95% CI (−0.189, −0.045)]. The *r*-value for the behavioral control group was 0.133 [95% CI (0.045, 0.218)].

**Table 4:** Test results of moderating effect.

Moderator variable	Subgroup^a^	*K*2	Intercept/Fisher’s *Z*	*r* ^b^	*F*	*Tau* ^2^	
			(95% CI)	(95% CI)		Level 2 variance	Level3 variance
Parental involvement type	AS	21	0.145 (0.092, 0.197)	0.144 (0.092, 0.194)	17.591^***^	0.005	0.007
	PyC	9	−0.118 (−0.191, −0.045)	−0.117 (−0.189, −0.045)			
	BC	6	0.134 (0.045, 0.222)	0.133 (0.045, 0.218)			
	CS	34	0.132 (0.084, 0.181)	0.131 (0.084, 0.179)			
Grade	Preschool	4	0.151 (−0.029, 0.331)	0.150 (−0.029, 0.319)	0.263	0.018	0.000
	Primary	15	0.120 (0.035, 0.206)	0.119 (0.035, 0.203)			
	Middle	26	0.081 (0.023, 0.138)	0.081 (0.023, 0.137)			
	Primary and middle	17	0.087 (0.018, 0.157)	0.087 (0.018, 0.156)			
	College	3	0.106 (−0.056, 0.269)	0.106 (−0.056, 0.263)			
Parental gender	Father	9	0.065 (−0.037, 0.167)	0.065 (−0.037, 0.165)	0.3	0.017	0.002
	Mother	14	0.096 (0.011, 0.182)	0.096 (0.011, 0.180)			
	Parental	47	0.108 (0.061, 0.155)	0.108 (0.061, 0.154)			
Region	East	51	0.085 (0.044, 0.126)	0.085 (0.044, 0.125)	0.098	0.017	0.000
	West	15	0.098 (0.021, 0.176)	0.098 (0.021, 0.174)			
Publication type	Journal	58	0.111 (0.066, 0.155)	0.111 (0.066, 0.154)	0.934	0.016	0.003
	Dissertation	12	0.062 (−0.029, 0.152)	0.062 (−0.029, 0.151)			

a The true Fisher’s *Z* value for each subgroup was obtained separately by using each subgroup as the reference group in turn in the three-level model.

b *r* values were transformed by Fisher’s *Z* value. ^***^p < 0.001.

#### 3.6.2 Grade

The moderating effect of grade was not significant (*F*_(4,60)_ = 0.900, *p* = 0.552). The *r*-values were 0.150 [95% CI (−0.029, 0.319)], 0.119 [95% CI (0.035, 0.203)], 0.081 [95% CI (0.023, 0.137)], 0.087 [95% CI (0.018, 0.156)], and 0.106 [95% CI (−0.056, 0.263)] for pre-school, elementary, middle school, elementary and secondary, and university, respectively. There were no significant differences between the *r*-values for the subgroups of grade.

#### 3.6.3 Parental gender

The moderating effect of parental gender was not significant (*F*_(2,67)_ = 0.300, *p* = 0.742). The *r*-values for maternal involvement and parental involvement were 0.096 [95% CI (0.011, 0.180)], and 0.108 [95% CI (0.061, 0.154)], respectively. The *r*-value for father involvement was 0.065 [95% CI (−0.037, 0.165)]. The *r*-values for the three subgroups of parental gender were not significantly different.

#### 3.6.4 Region

The moderating effect of region was not significant (*F*_(1,64)_ = 0.098, *p* = 0.756). The *r*-values were 0.085 [95% CI (0.044, 0.125)] and 0.098 [95% CI (0.021, 0.174)] for the Eastern and Western culture groups, respectively.

#### 3.6.5 Publication type

The moderating effect of publication type was not significant (*F*_(1,68)_ = 0.934, *p* = 0.337). The *r*-values was 0.111 [95% CI (0.066, 0.154)] and 0.062 [95% CI (−0.029, 0.151)] for the journal group and master’s thesis group, respectively.

## 4 Discussion

### 4.1 Main effect of parental involvement and students’ creativity

The present meta analysis integrated the contradictory findings from 37 independent primary studies uncovering a statistically significant positive correlation (*r* = 0.101) between parental involvement and students’ creativity scores. This result thereby affirms Hypothesis 1 which suggests that while previous empirical studies may exhibit variations, they still align with the predictions of EST (Bronfenbrenner and Morris, 2007; Woolfolk, 2013) and ESMCD (Yeh, 2004).

EST proposed that individual development is impacted by four interconnected systems—macrosystems, exosystems, mesosystems, and microsystems. Specifically, it suggests that within the microsystem, the family microenvironment may wield influence over children’s development (Bronfenbrenner, 1977; Bronfenbrenner and Morris, 2007; Woolfolk, 2013). The EST did not explicitly state that microsystem foster creativity, however, the creativity is an inner part of individual’s development. Therefore, it is reasonable to deduce that microsystem may affect the individual’s creativity.

This inference is supported by the ESMCD which states that the home environment established by parents within an intermediate system positively impacting creativity via three pathways: fostering independence, providing creative activity engagement, and directly encouraging participation in such activities (Yeh, 1999, 2004). Independence development can foster a child’s ability and character of braveness to try out, which is conducive to the creative development of adventure. Providing creative activity engagement can enable children to receive more love and encouragement, which is conducive to creating a constructive family atmosphere. This family atmosphere includes flexibility in family structure, caring for family members, mutual trust and support, opportunities to express emotions, and a high emphasis on cultural and intellectual activities, which contribute to the development of creative potential. Directly enveloping participation helps cultivate children’s problem solving ability and adaptive cognition skills, which play a key role in the creative development process. Through these influences, parents have a significant influence on shaping individual characteristics, and these creative personality traits later have a significant influence on creativity. Parents who adopt these influences also build a supportive home environment that contributes to daily creativity.

Furthermore, a recent empirical study presented some evidence supporting this claim. Jankowska and Gralewski (2022) found that a constructive parenting style (autonomy granting) was positively related to three of four factors of the climate for creativity in the parent–child relationships, i.e., encouragement to experience novelty and variety (*r* = 0.330), support of perseverance in creative efforts (*r* = 0.390), and encouragement to fantasize (*r* = 0.280).

### 4.2 Moderating effects of parental involvement type

This study discovered that parental involvement type significantly moderated the relationship between parental involvement and student creativity, supporting Hypothesis 2.

Parental autonomy support was significantly and positively correlated with student creativity (*r* = 0.144), a result that further explains the main effect and suggests that parental autonomy support is conducive to fostering student creativity. This is consistent with the facilitation perspective and is supported by similar studies (Kim, 2012; Moltafet et al., 2018).

The above result is also supported by SDT-related research. SDT suggests that parental autonomy support creates a home environment that develops children’s intrinsic resources (e.g., generates intrinsic motivation), which in turn influences children’s creative development (Deci and Ryan, 2012; Ryan and Deci, 2019, 2020).

Content support and students’ creativity scores were significantly positively correlated (*r* = 0.131), and the *r* value of this result was higher than the main effect, indicating that content support is more conducive to the promotion of students’ creativity. This is consistent with the facilitation view, and this result is also supported by a line of related studies (e.g., Cho and Lin, 2011; Oh et al., 2014). In other words, parents actively providing study guidance for their children may contribute to the fostering of students’ creativity.

Behavioral control was significantly correlated with students’ creativity scores (*r* = 0.133), indicating that behavioral control may promote the development of creativity in students. This is consistent with the facilitation view. This result is also consistent with the results of previous studies (Man et al., 2015; Ren et al., 2017; Chen et al., 2021). Through the strategy of behavioral control, parents may establish rules for their children’s daily activities and behaviors, fostering effective problem-solving skills and playing a protective role in adolescent development.

Psychological control was significantly negatively correlated with students’ creativity scores (*r* = −0.117), contrary to the results of the main effect, suggesting that psychological control may hinder the development of creativity in students. This is consistent with the results of the hindrance view (e.g., Liang et al., 2021) which says that psychological control leads to psychological harm that negatively affects creativity. This result suggests that not all parental involvement has a positive impact on creativity, and that psychological control, a parental involvement type, can weaken students’ intrinsic motivation and thus negatively affect their creative development.

### 4.3 Moderating effects of other variables

The present meta-analysis showed that the moderating effect of grade on the relationship between parental involvement and student creativity was not significant and did not support Hypothesis 3. This is inconsistent with the findings of previous studies (Barbot et al., 2016; Liang et al., 2021). There may be two reasons for this. Firstly, in the process of parental involvement in school, parental involvement is more likely to be achievement-oriented involvement than creative development-oriented involvement. Research suggests that excessive concern for academic performance may affect student creativity (Yi et al., 2013). Second, the level of parental involvement in schooling programs varies little across stages (Jeynes, 2012).

The present study demonstrated that the moderating effect of parental gender on the relationship between parental involvement and student creativity was not significant, supporting Hypothesis 4. This is inconsistent with the results of previous studies (Wang, 2018; Oh et al., 2014). The possible reasons for this came as following. First, the samples included in the present study is too small to tap the real differences. Second, although more involvement of mothers has been reported, parental involvement also needs to pass through other variables, such as student autonomy motivation, in order to have an impact on children’s creativity (Ryan and Deci, 2019, 2020).

The present study presented that the moderating effect of region on the relationship between parental involvement and student creativity was not significant and did not support Hypothesis 5. This is inconsistent with previous findings (Niu and Sternberg, 2001, 2002, 2003). A possible reason leading to this result may be that differences in the subtypes themselves in different regions lead to different results in different regions. For example, Yamamoto et al. (2022) observed the significant difference between Chinese and Japanese parental involvement suggesting that the East group should be divided into more subgroups. Dotterer (2022) noted that the variations in parental participation existed at the racial/ethnic level and the longitudinal effects of parental involvement on academic achievement which showed the significant differences could be observed among Asian Americans and other races, such as African Americans, European Americans, and Hispanics. Further research is needed to identify the reasons.

This study did not detect the moderating effect of publication type and did not support Hypothesis 6. This is inconsistent with the previous findings on the detection of publication bias (Card, 2012; Borenstein et al., 2009). The result indicated that the differences in publication type and publication bias in this study were negligible, which further enhances the credibility of the results of this study.

### 4.4 Theoretical and practical implications

The present study has theoretical significance:

(1) This is the first meta-analysis concerning on the relationship between parental involvement and creativity in the last 30 years. It integrates conflicting original studies which presented more reliable results.
(2) The results of this study reduces the gap between the empirical results about parental involvement and creativity and the theoretical predictions of ESMCD (Yeh, 1999, 2004).
(3) The results of this study revealed new problems deserving further investigation. That is, parental involvement needs to be systematically classified to clarify its connotation.

There are some implications for practice.

Firstly, an environment of independent support is conducive to the enhancement of the individual’s creativity. Therefore, in practice, parents provide autonomous support when raising their children by allowing them to make independent decisions on certain issues, or by listening to their children’s opinions and expressing their own opinions, which can promote children’s creativity. Moreover, parents avoid psychologically controlled participation in order to avoid harming their children’s creativity.

Secondly, it is very important to cultivate and protect internal factors of individuals. As emphasized by the ecosystem model of creativity, family and school experiences can only have positive effects without individual factors. The results show that from the SDT perspective, parental autonomy support and behavioral control can have a positive impact on creativity. Therefore, in practice, attention should be paid to protect students’ internal motivation, or promote the internalization of external motivation, to enhance their belief in creativity.

### 4.5 Research limitations and future prospects

By conducting a meta-analysis of the primary studies, the results of the current paper reaffirm the link between the empirical evidence and the predictions derived from EST (Bronfenbrenner and Morris, 2007) and ESM of creativity development (Yeh, 2004). At the same time, it reveals that not all parts of parental involvement are positively correlated with student creativity (i.e., negative effect of psychological control).

However, there are some limitations in this study. Firstly, the number of primary studies in some subgroup analyses is small. The small number of primary studies may make some subgroup analyses impossible (see Tables 2, 4).

Secondly, it failed to analyze the moderator effect of creativity measuring tools. These tools included (see Table 1) measured different aspects of creativity including creative thinking, creative problem solution, creative drawing production, domain creativity, scientific creativity, spatial-graphic creativity, creative activities, creative behaviors. Because of this diversity, the current meta-analysis did not provide analysis of the moderator effect of measures.

Thirdly, the interaction between parental involvement and other factors and their impact on student creativity deserve to be studied carefully. Given the modest correlation between these two variables, it suggests the potential influence of additional factors including individual factors (e.g., internal motivation, openness to experience, etc.), educational factors (e.g., parents’ expectations of their children’s creativity, balance between free exploration and necessary guidance, encouragement of students’ creativity, provision of rich learning resources, etc.), school factors (communication between parents, schools, and teachers), and societal factors (national policies to enhance students’ creativity, socio-cultural environment that emphasizes creativity). To explore the interaction between parental involvement and these factors, researchers can choose a specific factor, such as internal motivation, and build a mediation model of parental involvement, internal motivation and students’ creativity. Furthermore, it is also possible to select several factors to build more complex chain mediation models, mediation models with moderation, and so on.

Several issues should be further studied in the future.

First, much more primary studies are urgently needed. Some new primary studies should put emphasis on the parental control types, western cultures and high quality literature. The reason for the conflicting findings of the original literature included in this meta-analysis may be that the sample of the original study is not representative enough. When the samples of the original studies are merged together through meta-analytic methods, The sample size has been expanded, and the larger the sample size, the greater the reliability of our conclusion generalization (Card, 2012).

Second, future study should pay more attention to measurement of creativity. These measurement tools that focus on the product, press, processes of creativity is recommended in the future studies. Some studies using different measurements of creativity are needed when further exploring the relationship between parental involvement and individual creativity.

Third, future research should explore why the correlation between parental involvement and creativity is not high. In addition to parental involvement as a factor influencing creativity, other factors (e.g., individual factors) also have an impact on students’ creativity (van der Zanden et al., 2020). Bronfenbrenner (1977) and Yeh (2004) agreed that parental involvement is a meso-system in the overall ecological system and interacts dynamically with the micro-system and the macro-system. And a deduction can be made that their interaction could contribute to the development of individual’s creativity. However, the specific mechanism of this interaction has not been tested carefully in previous research.

Fourth, parental involvement may have an impact on student creativity through other variables. For example, Cho and Lin (2011) found that internal motivation and intellectual beliefs partially mediated the effects of positive parental involvement on creative problem solving in math and science. This implies that parental involvement should collaborate with other factors, such as internal motivation, in shaping creativity during its developmental process. That means that the creative children may elicit a different kind of parental involvement from their parents.

## 5 Conclusion

Based on this meta-analysis of 37 independent primary studies, a positive yet weak correlation is observed between parental involvement and student creativity. Also, parental autonomy support, content support, and certain types of parental behavioral control positively impact student creativity, whereas parental psychological control exhibits a negative association with student creativity. Furthermore, the correlation between parental involvement and student creativity appears to be minimally influenced by factors such as grade, parental gender, and region.

## Data availability statement

The original contributions presented in the study are included in the article/Supplementary material, further inquiries can be directed to the corresponding author.

## Author contributions

HF: Conceptualization, Funding acquisition, Methodology, Supervision, Writing – review & editing. YF: Data curation, Formal analysis, Methodology, Visualization, Writing – original draft, Writing – review & editing. YZ: Data curation, Resources, Writing – original draft.
