# Supplementary material for: Parental involvement and student creativity: a three-level meta-analysis
Source: Front Psychol. 2024 Sep 11;15:1407279. doi: 10.3389/fpsyg.2024.1407279 (PMC11422223; doi:10.3389/fpsyg.2024.1407279)
Supplement: Supplementary file 2 [file Data_Sheet_2.docx]

library("openxlsx")

library("metafor")

library("meta")

data1<-read.xlsx("parental involvement andstudent creativity. data.xlsx",colNames = TRUE)

full.model <- rma.mv(yi = z,

V = var.z,

slab = author,

data = data1,

random = list(~ 1 | es.id, ~ 1 | author),

test = "t",

method = "REML")

summary(full.model)

n <- length(data1$var.z)

list.inverse.variances <- 1/(data1$var.z)

sum.inverse.variances <- sum(list.inverse.variances)

squared.sum.inverse.variances <- (sum.inverse.variances)^2

list.inverse.variances.square <- 1/(data1$var.z^2)

sum.inverse.variances.square <- sum(list.inverse.variances.square)

numerator <- (n-1)*sum.inverse.variances

denominator <- squared.sum.inverse.variances - sum.inverse.variances.square

estimated.sampling.variance <- numerator/denominator

I2_1 <- (estimated.sampling.variance)/(full.model$sigma2[1]+full.model$sigma2[2]+estimated.sampling.variance)

I2_2 <- (full.model$sigma2[1])/(full.model$sigma2[1]+full.model$sigma2[2]+estimated.sampling.variance)

I2_3 <- (full.model$sigma2[2])/(full.model$sigma2[1]+full.model$sigma2[2]+estimated.sampling.variance)

amountvariancelevel1 <- I2_1 *100

amountvariancelevel2 <- I2_2 *100

amountvariancelevel3 <- I2_3 *100

amountvariancelevel1

amountvariancelevel2

amountvariancelevel3

publicationyear <- rma.mv(yi = z, V = var.z,

mods = ~ Publication.year,

random = list(~ 1 | es.id, ~ 1 | author),

tdist=TRUE, data=data1)

summary(publicationyear, digits=3)

quality assessment <- rma.mv(yi = z, V = var.z,

mods = ~ quality assessment,

random = list(~ 1 | es.id, ~ 1 | author),

tdist=TRUE, data=data1)

summary(质量评价, digits=3)

library(meta)

library(metafor)

m <-metagen(TE=data1$z,

seTE=data1$se.z,

studlab=paste(data1$es.id, data1$Publication.year, sep = "."),

sm="Fisher's Z",backtransf=TRUE)

summary(m)

#Rosenthal

fsn(yi=z,vi=var.z,type="Rosenthal",data=data1)

funnel(m)

#trimfill

trimfill(m)

m.trimfill<-trimfill(m)

funnel(m.trimfill,xlab ="Fisher's Z" )

col.contour = c( "black", "blue","darkslategray1")

funnel.meta(m.trimfill, xlim = c(-1, 1),

contour = c(0.9, 0.95, 0.99),

col.contour = col.contour)

#egger test

metabias(m,method.bias = "linreg",plotit = T,k.min=5)

#begg

metabias(m,method.bia="rank",plotit=T)#Minimum number of studies to perform test for funnel plot asymmetry.

generaldelinquency1 <- rma.mv(yi = z, V = var.z,

mods = ~ participationBC + participationPYC+participationCS,

random = list(~ 1 | es.id, ~ 1 | author),

tdist=TRUE, data=data1)

summary(generaldelinquency1, digits=3)

generaldelinquency1 <- rma.mv(yi = z, V = var.z,

mods = ~ participationAS + participationPYC+participationCS,

random = list(~ 1 | es.id, ~ 1 | author),

tdist=TRUE, data=data1)

summary(generaldelinquency1, digits=3)

generaldelinquency1 <- rma.mv(yi = z, V = var.z,

mods = ~ participationAS + participationBC+participationCS,

random = list(~ 1 | es.id, ~ 1 | author),

tdist=TRUE, data=data1)

summary(generaldelinquency1, digits=3)

generaldelinquency1 <- rma.mv(yi = z, V = var.z,

mods = ~ participationAS + participationBC+participationPYC,

random = list(~ 1 | es.id, ~ 1 | author),

tdist=TRUE, data=data1)

summary(generaldelinquency1, digits=3)

generaldelinquency1 <- rma.mv(yi = z, V = var.z,

mods = ~ Grades1primary + Grades2middle+Grades3中primary+Grades4collage,

random = list(~ 1 | es.id, ~ 1 | author),

tdist=TRUE, data=data1)

summary(generaldelinquency1, digits=3)

generaldelinquency1 <- rma.mv(yi = z, V = var.z,

mods = ~ Grades1preschool + Grades2middle+Grades3中primary+Grades4collage,

random = list(~ 1 | es.id, ~ 1 | author),

tdist=TRUE, data=data1)

summary(generaldelinquency1, digits=3)

generaldelinquency1 <- rma.mv(yi = z, V = var.z,

mods = ~ Grades1preschool + Grades1primary+Grades3中primary+Grades4collage,

random = list(~ 1 | es.id, ~ 1 | author),

tdist=TRUE, data=data1)

summary(generaldelinquency1, digits=3)

generaldelinquency1 <- rma.mv(yi = z, V = var.z,

mods = ~ Grades1preschool + Grades1primary+Grades2middle+Grades4collage,

random = list(~ 1 | es.id, ~ 1 | author),

tdist=TRUE, data=data1)

summary(generaldelinquency1, digits=3)

generaldelinquency1 <- rma.mv(yi = z, V = var.z,

mods = ~ Grades1preschool + Grades1primary+Grades2middle+Grades3中primary,

random = list(~ 1 | es.id, ~ 1 | author),

tdist=TRUE, data=data1)

summary(generaldelinquency1, digits=3)

generaldelinquency1 <- rma.mv(yi = z, V = var.z,

mods = ~ mother + parents,

random = list(~ 1 | es.id, ~ 1 | author),

tdist=TRUE, data=data1)

summary(generaldelinquency1, digits=3)

generaldelinquency1 <- rma.mv(yi = z, V = var.z,

mods = ~ father + parents,

random = list(~ 1 | es.id, ~ 1 | author),

tdist=TRUE, data=data1)

summary(generaldelinquency1, digits=3)

generaldelinquency1 <- rma.mv(yi = z, V = var.z,

mods = ~ father + mother,

random = list(~ 1 | es.id, ~ 1 | author),

tdist=TRUE, data=data1)

summary(generaldelinquency1, digits=3)

generaldelinquency1 <- rma.mv(yi = z, V = var.z,

mods = ~ Culturalwest,

random = list(~ 1 | es.id, ~ 1 | author),

tdist=TRUE, data=data1)

summary(generaldelinquency1, digits=3)

generaldelinquency1 <- rma.mv(yi = z, V = var.z,

mods = ~ Culturaleast,

random = list(~ 1 | es.id, ~ 1 | author),

tdist=TRUE, data=data1)

summary(generaldelinquency1, digits=3)

generaldelinquency1 <- rma.mv(yi = z, V = var.z,

mods = ~ Publicationtype2dissertation,

random = list(~ 1 | es.id, ~ 1 | author),

tdist=TRUE, data=data1)

summary(generaldelinquency1, digits=3)

generaldelinquency1 <- rma.mv(yi = z, V = var.z,

mods = ~ Publicationtype1journal,

random = list(~ 1 | es.id, ~ 1 | author),

tdist=TRUE, data=data1)

summary(generaldelinquency1, digits=3)

library(esc)

convert_z2r(0.101)
